# Clinical practice guidelines: sexual dysfunction in gynecological cancer patients

**DOI:** 10.1093/sexmed/qfaf0066

**Published:** 2025-09-04

**Authors:** Sharon Peleg Nesher, Mijal Luria, Gideon Sartorius, Francesca Tripodi, Michal Lew-Starowicz, Stephanie Both, Elisa Maseroli, Yacov Reisman, Giovanni Corona

**Affiliations:** Division of Oncology, Tel Aviv Medical Center, Tel Aviv, 6423906, Israel; Rotem Center - The Israeli Center for Sex Therapy, Jerusalem, 9626935, Israel; Rotem Center - The Israeli Center for Sex Therapy, Jerusalem, 9626935, Israel; Department of Obstetrics and Gynecology, Hadassah Hebrew University Medical Center, Jerusalem, 91240, Israel; Sexual Medicine, Women’s Clinic, University Hospital Basel, Basel, 4031, Switzerland; Institute of Clinical Sexology, Rome, 00198, Italy; International Online Sexology Supervisors (IOSS); Department of Psychiatry Center of Postgraduate Medical Education, Warsaw, 23100416, Poland; Department of Sexology and Psychosomatic Gynecology and Obstetrics, Amsterdam UMC, Amsterdam, 1105 AZ, The Netherlands; Andrology and Gender Endocrinology Unit, Careggi University Hospital, Florence, 50139, Italy; Department of Sexual Medicine, Flare-Health, Amsterdam, 1432BE, The Netherlands; Department of Sexual Health, Reuth Rehabilitation Hospital, Tel Aviv, 6772830, Israel; Endocrinology Unit, Azienda USL, Maggiore-Bellaria Hospital, Bologna, 40133, Italy

**Keywords:** gynecological cancer, sexual health, sexual dysfunction, cancer care, oncosexology

## Abstract

**Background:**

Sexual dysfunctions (SDs) due to gynecological cancer (GC) are common. Healthcare providers (HCPs) are often not prepared to address sexual health issues, missing the opportunity to provide comprehensive post-cancer survivorship care.

**Aim:**

To review the available evidence about diagnosing and managing SD after GC and providing practical clinical suggestions on behalf of the European Society of Sexual Medicine.

**Methods:**

A systematic literature search was performed on Pubmed and Medline for the relevant literature from January 1980 until June 2024.

**Outcomes:**

Recommendations were provided according to the Oxford Centre for Evidence-Based Medicine 2011 Levels of Evidence criteria, focusing on clinical practice.

**Results:**

The main areas covered include the impact of diagnosis and treatment of GC (surgery, chemotherapy, immunotherapy, and radiotherapy) on sexual health; the process of screening, counseling, and referral; medical and psychological management of SD; issues related to special populations, ie, sexual minorities and previvors.

**Clinical Implications:**

Addressing aspects of sexual health is important in patients with GC during diagnosis, treatment, and post-cancer care. Diagnosis and treatment of SDs should follow the recommendations in non-cancer patients, but specific aspects linked to cancer and its treatment should be kept in mind.

**Strengths and Limitations:**

All studies have been evaluated by a panel of experts who provide comprehensive, evidence-based recommendations for clinical practice.

**Conclusion:**

HCPs should feel comfortable addressing sexual health topics in patients with GCs due to the abundance of available data. Appropriate sexological interventions can improve the quality of life for patients and their partners.

## Introduction

The prevalence of bothersome sexual dysfunctions (SDs) among gynecological cancer (GC) survivors approaches 90% compared to 40% among the general female population.^1^ According to the available literature, SD can lead to distress and significantly impact patients’ and their partners’ quality of life (QoL). Attention to survivorship and QoL is crucial to patients’ comprehensive care, and sexuality serves as an important indicator of QoL and overall survival.^2^ Concerns about sexual side effects can impact adherence to cancer treatments or delay risk reduction surgery recommendations, as in the case of previvors.^3^

A large body of evidence clarified that patients request their health professionals to pay attention to sexual concerns, and demand to receive information about treatment consequences for sexual functioning, as well as practical advice on dealing with dysfunctions.^4–8^ Recent data showed that up to 50% of patients interviewed would use sexology treatment facilities if their oncologist recommended them. In particular, a consultation with a health care physician dealing with SD was the preferred form of request advanced (45%-82%), followed by a psycho-sexologist (36%-65%), nurses (24%), couple therapy (29%-53%), and support groups (12%-32%).^9–11^

According to the current National Comprehensive Cancer Network guidelines, it is important to assess sexual health in every woman at the time of diagnosis, during treatment, after treatment completion, and in longer-term survivorship.^12^ Although many healthcare providers (HCPs) recognize the importance of addressing sexual health for GC patients,^11^^,^^13^^,^^14^ available data indicate that sexual health is still a neglected topic in cancer care for several reasons.^7^^,^^11^^,^^15^^,^^16^ Patients often report that feelings of discomfort preclude them from openly discussing sexuality with their HCPs.^4^^,^^6^^,^^8^^,^^17^ On the other hand, HCPs recognize several perceived barriers such as time constraints, culture/religion issues, embarrassment, lack of knowledge and training, and lack of resources to provide an adequate support if needed.^14^^,^^15^^,^^18^ In addition, so far, comprehensive, evidence-based clinical practice guidelines are scarce.

This article, based on behalf of the European Society for Sexual Medicine (ESSM), aims to summarize the available literature on sexual health in GC patients and to provide practical clinical suggestions and statements to manage this issue correctly.

## Methods

### Search strategy

A comprehensive PubMed, MEDLINE, and Cochrane Library search was conducted based on the following keywords: “gynaecological cancer” AND “Sexual relationship,” “gynaecological cancer” AND “Sexuality,” “gynaecological cancer” AND “Psychosexual issues,” “gynaecological cancer” AND “Patient-reported outcomes,” “gynaecological cancer” AND “Quality of life,” “gynaecological cancer” AND “Radiotherapy,” “gynaecological cancer” AND “Chemotherapy,” “ovarian cancer” AND “Sexual relationship,” “ovarian cancer” AND “Sexuality,” “ovarian cancer” AND “Psychosexual issues,” “ovarian cancer” AND “Patient-reported outcomes,” “ovarian cancer” AND “Quality of life,” “endometrial cancer” AND “Sexual relationship,” “endometrial cancer” AND “Sexuality,” “endometrial cancer” AND “Psychosexual issues,” “endometrial cancer” AND “Patient-reported outcomes,” “endometrial cancer” AND “Quality of life,” “cervical cancer” AND “Sexual relationship,” “cervical cancer” AND “Sexuality”, “cervical cancer” AND “Psychosexual issues,” “cervical cancer” AND “Patient-reported outcomes,” “cervical cancer” AND “Quality of life,” “vulvar cancer” AND “Sexual relationship,” “vulvar cancer” AND “Sexuality,” “vulvar cancer” AND “Psychosexual issues,” “vulvar cancer” AND “Patient-reported outcomes,” “vulvar cancer” AND “Quality of life”. Studies from January 1980 to June 2024 were included. A total of 573 full-text articles were found. The final selection of papers was based on the clinical and research expertise of the committee members. The search was restricted to full-text articles written in English.

Recommendations, where possible, were given according to the Oxford 2011 Levels of Evidence criteria.^19^ Specific statements are provided based on literature and deliberations among a board of experts, identified on behalf of ESSM Scientific Committee and ESSM Board.

## Impact of gynecological cancer on sexual health

***Statement #1:** The diagnosis and treatment of gynecological cancer (GC) impact on patients biological, psychological, and social determinants, leading to sexual health impairment. A multidisciplinary approach is recommended. (Good clinical practice)*

***Statement #2:** Depression/anxiety, fear of recurrence, or anticipation of sexual pain often result in low sexual desire in GC survivors. (LoE 3, Grade C)*

***Statement #3**: Surgery, chemotherapy, and radiotherapy may cause genital arousal disorder, orgasmic dysfunction, and genito-pelvic pain/penetration disorder through a multi-modal mechanism involving hormonal, vascular, and neural disruptions. (LoE 1 Grade A)*

***Statement #4:** Iatrogenic premature ovarian insufficiency (POI) is associated with a higher risk of SD than natural menopause. (LoE 1 Grade 1)*

### Evidence

A recent study using the Diagnostic and Statistical Manual of Mental Disorders, Fifth Edition criteria for the estimation of SD prevalence in GC survivors 1 year after treatment found that 70.9% had sexual interest/arousal disorder, 60.0% had genito-pelvic pain/penetration disorder, 20.0% had orgasmic disorder, whereas 43.6% reported more than one disorder.^20^ In addition, 65.1% declared decreased sex frequency as compared to pre-treatment.^20^ Finally, decreased sexual satisfaction was also noted in 52.2% of sexually active women.^20^

Lack of interest in engaging in sexual interactions could be secondary to depression/anxiety, fear of recurrence, or anticipation of sexual pain (or any other type of non-pleasurable touch). Women taking antidepressant medication such as selective serotonin reuptake inhibitors (SSRIs) for depression/anxiety or hot flashes may have a further decline in sexual desire, sexual arousal dysfunction, and orgasm difficulties.^21^^,^^22^

Several mechanisms may affect the genital arousal response in GC survivors, including hypoestrogenism-induced atrophy, injury or compression to pudendal and/or pelvic nerves, peripheral neuropathy, myofascial and musculoskeletal injury, lymphedema, intestinal injury, and urinary tract injury.^23^ These processes result in poor lubrication and pain during sexual activity.

In addition, pelvic floor dysfunction (PFD) is a prevalent and multifaceted issue among survivors of GC, frequently manifesting as urinary and fecal incontinence, pelvic organ prolapse, dyspareunia, and SD, significantly impairing QoL.^24^ In a recent systematic review, it was reported that SD is highly prevalent among GC survivors with PVD, the rates of dyspareunia ranging from 12% to 58% in cervical cancer survivors and 7% to 39% in endometrial cancer survivors.^25^

#### Surgery

Surgical procedures can have an impact on autonomic nerves and, therefore, impair arousal and orgasmic function. Radical hysterectomy (RH) may cause pelvic floor nerve damage leading to lymphedema, genital numbness, lack of lubrication, and vaginal shortening.^26^ Loss of bladder sensation and detrusor voiding activity may also occur.^27^ Orgasmic disorder has been reported as a consequence of RH^28^^,^^29^; however, its prevalence decreases with time, with no difference between patients and controls 12 months after intervention or later.^29^^,^^30^ Other studies have reported no effects of hysterectomy on orgasmic function.^31^^,^^32^ In the last few years, the use of laparoscopic and robotic approaches has become more common, improving postoperative outcomes and QoL.^33^^,^^34^

Treatment of vulvar intraepithelial neoplasia or vulvar cancer ranges from local vulvar excision to radical vulvectomy, which can include the removal of the entire vulva, regional lymph nodes, and, in some cases, the clitoris. The radicality of the surgery is associated with poorer sexual function,^35^ and clitoridectomy is often associated with inability to experience orgasms.

Among surgery procedures, bilateral salpingo-oophorectomy, a standard practice for many GCs, may induce or worsen menopausal symptoms due to both testosterone and estrogen defects.^36^ Iatrogenic menopausal symptoms include hot flashes and night sweats, sleep disturbances, irritability, anxiety, depression, cognitive changes, vaginal dryness, painful intercourse, and urinary symptoms. In addition to atrophy, hypoestrogenism causes a shift of the vaginal microbiome, with consequent increase in vaginal pH, local immune changes, and increased cytokine synthesis, worsening vaginal dryness and burning.^37^ Usually, menopausal symptoms triggered by cancer treatment are more abrupt, intense, and/or prolonged than natural menopause.^36^^,^^38^

#### Chemotherapy

Among the chemotherapy agents most commonly used in the treatment of GC are Taxanes and Platinum agents. Both have been associated to peripheral neuropathy in different series, with a relevant incidence (30%-100% of cases).^39–41^ The development of neuropathy can play a detrimental impact on sexual functioning, leading to pain and hypersensitivity in the vagina, clitoris, and labia, with a detrimental effect on arousal and orgasmic function.^42–44^ Despite these consequences, the most feared side effect of chemotherapy is related to premature ovarian insufficiency, a significant risk factor for cancer-related SD.^45^

#### Immunotherapy

Immunotherapy may indirectly affect sexual function, mostly through autoimmune suppression of gonadal function.^46^ Indeed, hypophysitis, or inflammation of the pituitary gland, is considered the most common ICPi (immune checkpoint inhibitors)-related endocrinopathy, together with thyroid dysfunction.^47^ Immune-mediated neuropathy following immunotherapy may also contribute to SD.^48^^,^^49^

#### Radiotherapy

Radiotherapy can induce early superficial or more deep damages of the wall of all genital structures including vulvar skin, vagina, bladder, and rectum.^50^ Long-term effects include atrophy and fibrosis, resulting in pain and SD.^51–53^ Some studies have suggested a relative risk of orgasmic dysfunction of approximately 1.5 after radiation therapy.^54–56^

Regardless of the treatment type, the journey following a GC diagnosis is frequently marked by significant psychosocial distress, profoundly impacting survivors’ QoL.^36^^,^^57^^,^^58^ This distress is multifaceted, stemming not only from the existential threat of the disease, including pervasive death anxiety, fear of recurrence, and feelings of helplessness and hopelessness, but also from the tangible loss of physical function and altered body image.^5^^,^^39^ The emotional sequelae are often compounded by the rigorous and invasive nature of treatments, which can leave lasting physical and psychological scars. Systematic reviews continue to highlight the high prevalence of anxiety, depression, and cancer-specific distress among this population, underscoring the need for attentive psychosocial care.^59^^,^^60^

Beyond these intrapsychic challenges, the disease and its treatment often precipitate considerable distress across various life domains. Partner relationships can be significantly strained due to changes in intimacy, communication difficulties, and shifts in caregiver-patient roles. The return to, or maintenance of, employment can be fraught with challenges, including managing treatment side effects, cognitive changes (“chemo brain”), and potential workplace discrimination or lack of understanding. Furthermore, survivors may experience distress navigating the medical system, feeling overwhelmed by appointments, or perceiving a lack of empathetic communication from HCPs. Restrictions in everyday life activities due to fatigue, pain, or physical limitations, alongside a potential loss of “recovery space” (a safe, private environment conducive to healing), further contribute to a diminished sense of well-being and increased distress.^61^

Given the profound impact of these stressors, the role of robust social support, including a trusting and communicative physician-patient relationship, cannot be overstated. Studies consistently demonstrate that perceived social support acts as a crucial buffer against psychosocial distress and is positively associated with better adjustment and QoL in cancer survivors.^59^ A supportive healthcare environment, where patients feel heard, understood, and validated by their oncology team, is a fundamental component of this support system. Therefore, it is an essential aspect of basic psychosocial healthcare to systematically assess for these diverse stressors, emotional, relational, occupational, and systemic.^5^^,^^57^^,^^59^ This assessment, followed by the provision of basic supportive interventions such as empathetic listening, information provision, and timely referral to specialized services when indicated, forms a general, humanistic approach to care. This sensitive and multidisciplinary support, distinct from specific psychotherapeutic techniques, is integral to holistic patient management and should be a standard component of GC survivorship care.^6^^,^^36^^,^^57^^,^^59^^,^^60^

## Information, screening, counseling, and referral

***Statement #5**: We suggest carefully assessing and addressing sexual dysfunctions in genital cancer patients and survivors, providing individualized evaluation and treatment based on the patient’s preferences. (LoE 3, Grade B)*

***Statement #6**: The patient and, whenever possible, her partner need to be aware of the possible sexual difficulties due to the disease, and they should receive adequate information about possible sexual side effects of the treatments. (LoE 4, grade C)*

***Statement #7**: All gynecological cancer patients and survivors, and, whenever possible, their partners should be offered brief education and psychosexual counseling and when needed referral to a specialist. (Expert Opinion)*

### Evidence

Available data strongly support the need for HCPs to recognize the high incidence of SD among all patients diagnosed with GC and survivors. Research indicates that patients who are interested in sex and remain sexually active tend to have better survival rates compared to those who are not interested in sex and are not sexually active.^2^ HCPs should also provide detailed information regarding the anticipated physical alterations resulting from the cancer or its treatment, and their possible repercussions on sexual function.^62^

Acknowledgement, information, and screening of all patients for sexual function and satisfaction should be offered before the beginning of treatment and again at varied points of treatment and survivorship. A culturally sensitive approach, bearing in mind, the patient’s literacy level, cultural/religious beliefs, sexual orientation, and gender identity, is strongly advised. If the patient wishes it, it is appropriate to involve the partner in the care process.^63^

Counseling services should be adjusted to meet the specific needs of individuals in different age groups. Young adult GC women may report practical barriers (financial, time consumption, etc.), as well as emotional avoidance, whereas postmenopausal women may express difficulties facing stigma, shame, and discomfort.^6^

Counseling services can be provided by various HCPs who are experts in the field. Examples include nurse practitioners, clinical nurse specialists, social workers, psychologists, as well as patient groups or organizations that offer peer support. Nurses play a critical role in the multidisciplinary care of women with GC, addressing both oncologic and survivorship needs. As part of the multidisciplinary team, specialist nurses, such as a nurse midwife or any advanced practice registered nurses who are trained to assess, diagnose, and manage patient care, including prescribing medications and performing specific procedures, provide individualized, holistic care that encompasses physical, psychological, and sexual health challenges faced by patients, particularly in the context of treatment-induced menopause. They lead and coordinate menopause services, manage complex symptom profiles, and support the GC patient and her partner throughout the treatment and follow-up process, contributing to care planning alongside oncology, gynecology, and mental health professionals.^64^

Evidence from randomized trials supports the effectiveness of nurse-led interventions, such as those based on positive psychology, in improving sexual function, reducing depression, and enhancing well-being among cervical cancer survivors.^65^ Furthermore, national guidelines emphasize the necessity for nurses managing GC to receive specific training in menopause care, ensuring informed decision-making regarding Hormonal Replacement Therapy (HRT) and alternative treatments.^66^

Referral to further evaluation and treatment may be directed to a sexual medicine-trained physician and/or to a sex therapist.^21^ A comprehensive physical examination by a physician with expertise in genito-pelvic examination skills, knowledge of cancer pathophysiology, and the ability to collaborate with the patient’s treating oncologist is strongly suggested.^63^

#### Medical management of sexual problems

After therapeutic objectives have been established, according to contributing biopsychosocial factors, motivation, and aim of treatment, a therapeutic plan should be proposed and shared with the patient or the couple. Decision-making should take into account the individual risk-to-benefit ratio, life expectancy, patient and couple preference, and the fact that several medical treatments are off-label also in cancer-free patients.

In the following section, available treatments will be discussed in detail.

### Desire, arousal, and orgasm difficulties

***Statement #8**: Hormonal treatments (including transdermal testosterone) are not recommended in patients with a history of hormone-dependent neoplasia. (LoE1, Grade A)*

***Statement #9**: Approved hormonal and non-hormonal treatments for Hypoactive Sexual Desire Disorder (HSDD) have not been extensively studied in GC survivors, and more research is required. (Expert Opinion)*

#### Evidence

Cancer patients and survivors with low sexual desire complaints do not strictly meet the diagnostic criteria for Hypoactive Sexual Desire Disorder (HSDD) due to a situational condition associated with oncologic diagnosis and treatments. Nevertheless, common therapeutic options may be appropriate. Sometimes the empiric use of multiple strategies can be required.

Transdermal testosterone (T) treatment is considered as a safe and effective therapy in postmenopausal women without a history of hormone-dependent neoplasia.^67^ In these low-risk patients, T treatment, achieving blood concentrations of T that approximate premenopausal physiological ones, can be considered after a full clinical assessment has been performed and other factors contributing to SD have been addressed.^67^ However, T is not commercially available as a treatment for female HSDD in most countries, including Europe and the United States. On the other hand, GC survivors were excluded from RCTs investigating T therapy, and given the lack of safety data, systemic T treatment is not recommended in these women.^67^

As for non-hormonal treatments, two medications are approved in the United States for the treatment of HSDD in premenopausal women: flibanserin and bremelanotide. Although these compounds have a non-hormonal mechanism of action for improving libido, they have not been extensively studied in GC survivors, and additional research is needed in this population.^68^^,^^69^ Some data support the off-label use of the antidepressant bupropion in GC survivors with depression and associated sexual concerns. Nevertheless, indirect evidence suggests that bupropion can affect the metabolism of tamoxifen, and it should not be used in those women undergoing this adjuvant treatment.^70^

In GC survivors, Female Orgasm Disorder may be a consequence of dyspareunia, cancer treatment, use of antidepressants, and medical comorbidities, including neuropathy. After addressing vaginal dryness (see dedicated paragraph), sexual devices such as vibrators and clitoral vacuum stimulation devices can support oxygenated genital blood flow and enhance genital stimulation and arousal.^71^ Although they are theoretically safe in GC women, it should be considered that there are no safety regulations for the manufacture of these devices. Similarly, local (ie, alprostadil) and systemic (ie, phosphodiesterase type 5 inhibitors) vasodilation agents have been proposed to treat Female Arousal and Orgasm disorder based on a few controlled studies.^72^^,^^73^ They have a non-hormonal mechanism of action, but they have not been studied specifically in GC women; therefore, caution and evaluation of the risk of side effects are mandatory in this population.

## Vasomotor symptoms

***Statement #10:** Hormone Replacement Therapy (HRT) is the most effective treatment for menopausal vasomotor symptoms (VMS) and genitourinary syndrome of menopause. (LoE1, Grade A)*

***Statement #11:** Selective serotonin reuptake inhibitors (SSRIs), selective serotonin-norepinephrine reuptake inhibitors (SNRIs) or clonidine represent effective non-hormonal alternatives for vasomotor symptoms when HRT is contraindicated.(LoE1, Grade B)*

### Evidence

For women with early menopause (natural, surgical, or treatment-induced) without contraindications for estrogen use, HRT is recommended until at least the median age of menopause (51 years). Longer duration of HRT should be considered according to clinical condition and patient needs. Women without a uterus should receive estrogen alone; for women with a uterus, progestogen or bazedoxifene must be added for endometrial protection.^74^

Although HRT is not recommended in women with granulosa cell tumors or endometrial stromal sarcomas, studies of women with a history of epithelial ovarian, endometrial, and cervical cancers have not shown any detrimental effects.^75^

For women with prior estrogen-sensitive cancers, the use of non-hormonal treatment should be recommended first. SSRIs and selective serotonin-norepinephrine reuptake inhibitors (SNRIs) are effective non-hormonal alternatives for vasomotor symptoms.^76^^,^^77^ However, potential sexual side effects of SNRIs and SSRIs have to be discussed with the patient.^78^ Gabapentin, pregabalin, and clonidine may also decrease the frequency of hot flashes, although efficacy studies with these medications are limited.^76^^,^^77^ It should be emphasized, as mentioned earlier, that there may be potential negative interactions with tamoxifen.^70^ If non-hormonal treatment is not successful in relieving bothersome symptoms, HRT should be considered in conjunction with the patient’s oncologist.^75^

## Vaginal dryness

***Statement #12:** Vaginal lubricants and moisturizers relieve vaginal discomfort and pain during intercourse for women with mild to moderate vaginal dryness. (LoE3, Grade B)*

***Statement #13**: For bothersome vulvovaginal or urinary symptoms not relieved with non-hormonal therapies and with no contraindications, the use of vaginal hormonal treatment is recommended. (LoE1, Grade A)*

### Evidence

Vaginal moisturizers ease day-to-day discomfort, mimicking natural secretions. They are applied regularly, from every day to every 2-3 days, adhere to the vaginal lining, and maintain moisture and acidity.^79^ Lubricants provide short-term relief and are applied to the vagina, vulva, and partner’s penis before and during sexual activity.^79^ A wide variety of lubricants are available: water, silicone, mineral, hyaluronic acid vaginal gel, and plant oil-based, and some are not within the recommended osmolality and pH ranges.^79^ The specific analysis of their mechanisms is beyond the aim of the present article and revised elsewhere.^79^

For bothersome vulvovaginal or urinary symptoms (urinary urgency, recurrent urinary tract infections) not relieved with non-hormonal therapies and without contraindications for the use of systemic HRT, low-dose vaginal estrogen therapy,^80^^,^^81^ or other therapies (such as intravaginal dehydroepiandrosterone [DHEA]^82^ or ospemifene^83^) are recommended over systemic HRT. For women with early endometrial cancer who have completed successful treatment, including hysterectomy, low-dose vaginal estrogen therapy can be considered if non-hormone options are not successful.^74^^,^^84^ Low-dose topical DHEA improves vaginal dryness and sexual pain,^82^^,^^85^ providing local formation of sex steroids in peripheral tissues without significant changes in serum concentrations of estrogens or testosterone.^86^

Topical vaginal T has been found to improve signs and symptoms of vaginal atrophy without increasing serum estradiol or T levels, but large-scale, randomized, controlled trials are still lacking.^87^^,^^88^ Ospemifene is an oral selective estrogen receptor modulator approved by the Food and Drug Administration (FDA) for postmenopausal vaginal dryness and dyspareunia and in the European Union for use after breast cancer.^89^

Three energy-based therapies have been proposed: fractional microablative CO_2_ laser, erbium:YAG laser, and temperature-controlled radiofrequency. The data appear to be encouraging, but good evidence is still lacking.^90–92^ There is no data on long-term complications and specific concern about laser activation of fibroblasts, which may lead to fibrosis, stenosis, and scarring.^93^

## Vaginal stenosis and pelvic floor dysfunction

***Statement #14**: To prevent and/or delay vaginal adhesions, tightening, and shortening, we recommend using vaginal dilators. (LoE 3, Grade B)*

***Statement #15**: If penetration is painful or not pleasurable, non-penetrative sex should be explained, normalized, legitimatized, and supported. (Expert opinion)*

***Statement #16**: In the presence of pelvic floor dysfunction, we suggest the use of physical therapy with external and internal (intravaginal or intrarectal) manual therapies, including soft tissue mobilization, myofascial release, biofeedback, electrical stimulation, and therapeutic exercises. (LoE 2, Grade B)*

### Evidence

Vaginal stenosis is a common consequence of vaginal and pelvic radiotherapy, ranging from 50% to 80%.^94^ The problem usually appear in the first 5 years after radiotherapy, but late manifestations can evolve over several years after the treatment.^94^ The main risk factors for this side effect include dose and volume of vagina irradiated and irradiation to the lower portion.^94^

Evidence suggesting that routine use of dilators leads to improved sexual function is mainly supported by non-randomized or observational studies, and therefore inconclusive. Nevertheless, in clinical practice, this approach is recognized as an important tool in maintaining vaginal and sexual function after GC treatment, safely preventing or delaying vaginal stenosis.^95^ In an open-label RCT, after radiotherapy due to cervical cancer, there was a reduction in vaginal volume in all treatment groups analyzed, with no significant difference between them. However, women who used vaginal dilators had a lower frequency and severity of vaginal stenosis assessed by the Common Criteria for Adverse Events Version 3.0 scale after 1 year of treatment.^96^ A moderately sized prospective study found that patients with cervical cancer who maintained compliance with vaginal dilation treatment for more than 1 year experienced a reduced incidence of any vaginal stenosis (21% vs 39% in non-compliant patients).^97^ Additionally, a large single-site retrospective study demonstrated that patients with high dilator compliance had lower rates of functionally limiting vaginal stenosis at a 3-year follow-up (16% vs 45% in non-compliant patients).^98^ The last Cochrane Systematic Review on the topic dates back to 2014 and concluded that, in the absence of strong evidence from RCTs, several observational studies suggest that frequent dilation is associated with lower rates of self-reported stenosis; nevertheless, it has to be noted that RCT methodology is particularly challenging in this area.^99^

If penetration is painful or not pleasurable, sex therapy can introduce couples to a range of non-penetrative activities that enhance closeness and sexual satisfaction, such as sensate focus exercises and mutual masturbation. This promotes relaxation and connection, helping to decrease the physical and psychological tension that contributes to pain. Although data in patients with vaginal stenosis are lacking, non-penetrative sex has been reported to increase satisfaction in other populations, such as women with vaginismus.^100^  Box 1 reports the proposed management of vaginal stenosis related to GC treatment.

Box 1 **Proposed management of vaginal stenosis related to GC treatment.**

Based on the available data,^101–103^ we recommend:

Inform

1. To give information about the rationale for dilation therapy to all women at risk.

The rationale is to prevent vaginal adhesions, make future exams more manageable, and reduce fear of bodily changes and sexual activity.

2. To advise that a small amount of bleeding or “spotting” after dilator use is normal. A clinician should be contacted if there is a lot of new bleeding or pain.

Timing

3. Introduce the dilator early on in treatment. Be aware and sensitive to emotional responses.

4. The radiation oncologist should provide the first introduction before radiotherapy, and the oncology nurse should provide more extensive information during the first follow-up appointment.

5. Address to begin dilation approximately 2 weeks post radiotherapy when the acute inflammatory response has settled.

6. Advise the patient to discuss with her gynecologist after a follow-up physical examination the possibility of discontinuing dilation therapy when it is no longer required.

7. If stenosis develops, record toxicity using a recognized score. (Grade 1 no stenosis, grade 2 stenosis of the upper third of the vagina, grade 3 stenosis of more than the upper third).

Duration and frequency

8. Duration and frequency of dilation may range from three minutes twice a week up to 30 min daily for the first 6 months, once a week thereafter, and then occasionally after a year if not experiencing difficulty.

^*^There is strong evidence to support any recommended technique over another.

The technique

9. Advice the patient to start with the smallest dilator and progress to whatever size is comfortable.

10. It is best to insert vaginal dilators as deep as possible and to move the dilator around when inserted.

The dilators

11. Intravaginal acrylic cylinders may be used. The diameter and length of the cylinder may be adapted to women’s vaginal dimensions measured at each follow-up visit. If the vaginal dimensions change, the dilator may be replaced by one of the appropriate sizes.

12. Dilators need to be particularly clean; it is advisable to wash them and store them in a clean bag or case. They can be cleaned with soap and water, making sure they are rinsed thoroughly.

Use of lubrication

13. Use water-based lubricants with no perfumes or colorings.

Partner’s involvement

14. Whether or not the partner is actively involved should depend on the patient’s needs.

15. Enhance dilator accessibility and psychoeducational resources for supporting vaginal dilator use.

16. Ensure consistent institutional practice when introducing the dilator.

17. The availability of an informational brochure and Web site is desirable.

PFD shows an increased prevalence in women who have received treatment for GC, and it can contribute to poor sexual function, directly or via urinary/bowel incontinence, and pelvic organs prolapse.^104^ Moderate-level evidence supports the use of physical therapy, including external and internal (intravaginal or intrarectal) manual therapies, soft tissue mobilization, myofascial release, biofeedback, electrical stimulation, and therapeutic exercises with the aim of improving PFD symptoms in GC survivors. The strongest evidence concerns the positive role of pelvic floor muscle training in urinary incontinence^105^ and symptomatic prolapse.^106^ A recent meta-analysis of the sexual function outcomes after pelvic floor muscle training, combined with counseling and yoga or core exercises,^107^^,^^108^ found a significant improvement compared with controls in GC survivors.^109^

## Other gynecology related issues

### Fertility counseling

***Statement #17:** Fertility preservation techniques should be discussed, offered, and if interested, measures taken before starting treatment. (LoE 3, Grade B)*

#### Evidence

Fertility preservation techniques should be discussed with patients before the start of gonadotoxic therapies since chemotherapy and radiotherapy might disrupt reproductive function. The intensity of this deleterious impact depends on the patient’s age and the type and doses of chemotherapy/radiotherapy involved.^110^ Fertility preservation techniques include vitrification/cryopreservation of unfertilized oocytes, embryo cryopreservation with or without ovarian stimulation, or cryopreservation of the ovarian cortex.^111^ If no fertility preservation measures have been taken, counseling should be offered to deal with the consequences of infertility.^112^ The counseling process is complex since patients may have to decide under pressure. An online decision-aid tool might be helpful to facilitate the decision-making process.^113^

### Weight management

***Statement #18:** We suggest providing adequate information to maintain normal weight and avoid weight gain. (Expert Opinion)*

#### Evidence

Regular exercise, a healthy diet, and weight management should be addressed in all cancer survivors. Physical activity and exercise have beneficial effects on health-related QoL domains in cancer survivors, including fear of recurrence, body image/self-esteem, emotional well-being, sexuality, sleep disturbance, social functioning, anxiety, fatigue, and pain.^114^ It can be helpful for patients with symptoms of cognitive dysfunction and lymphedema.^75^ In people dealing with GC, it may decrease symptom burden.^115^ Accordingly, it increases sexual interest and overall sexual health, in endometrial cancer survivors.^116^

### Psychological management of sexual problems

***Statement #19**: Sexual rehabilitation interventions should take into consideration psychosocial factors when addressing the sexual care needs of individual patients and couples. (LoE 3, grade C)*

***Statement #20:** Structured individual or group interventions, preferably implementing educational, cognitive-behavioral, and mindfulness modules, are recommended for highly motivated, clinically stable GC survivors who want to engage in sexual activity. (LoE 4, grade C)*

#### Evidence

Only a few studies tested psychological interventions addressing sexual concerns for GC survivors, and the interpretation of results is largely limited due to heterogeneous samples investigated, selection bias, diversity and complexity of interventions, and lack of or short follow-up of the effects on sexual function, satisfaction, and relationship. Nevertheless, available evidence suggests that incorporating psychological support into sexual rehabilitation interventions for GC survivors enhances their ability to adapt to physical changes, process emotional challenges, and regain a sense of sexual well-being.^59^ Psychological factors with an impact in post-cancer sexual life adjustment include changes in body image and sexual self-schema, women’s attitudes and perceptions toward their own sexuality, the quality of their pre-existing relationship, their partner’s health status, the frequency of engaging in sexual activity, the ability to communicate effectively, and partner beliefs and differing perceptions.

Brief education and counseling alone were reported to improve the knowledge of sexual issues related to GC. Although it had no significant or just a transient positive influence on sexual function per se and communication within the relationship, related distress was decreased.^60^^,^^117^^,^^118^ Internet-based education and counseling also show a promising perspective and an easily accessible alternative to on-site interventions.^119–121^ Complex, brief interventions consisting of education, counseling, relaxation, mindfulness, and CBT techniques were found to increase sexual function (including desire, arousal, orgasm, and satisfaction) in some uncontrolled studies.^122^^,^^123^

Psychosexual education focused on changes related to cancer and rehabilitation strategies combined with the use of dilators, lubricants, and pelvic floor exercises improved sexual function, confidence about future sexual activity, and decreased vaginal health concerns in female breast, gynecological, and colorectal cancer survivors.^124^ Controlled studies on GC survivors and data from non-homogenous populations (breast and GC) show a beneficial effect of addressing intimacy and partner communication on relationship adjustment and sexual satisfaction.^125^^,^^126^ Well-powered RCTs on representative groups of GC survivors are needed to confirm the efficacy and stability of the effects of sexual intimacy and relationship-oriented interventions.

## Special populations

### Sexual and gender minority populations

***Statement #21:** We recommend that all HCPs enhance their cultural competence for sexual and gender minority in order to ensure the needs of these people better and to improve their health and sexual outcomes, including in the context of GC*. *(Expert Opinion)*

#### Evidence

In the last few years, sexual and gender minority populations have been identified as a diverse group at risk for receiving suboptimal care throughout the cancer care continuum.^127^ HCPs do not routinely ask patients about their sexual orientation or identity,^128^ and patients fear disclosure may lead to inappropriate treatment by the medical personnel.^129^

Lesbian and bisexual women may have higher rates of cervical cancer risk factors and behaviors compared with heterosexual women, including higher body mass index scores and smoking history.^130^ Many of them and/or their partners have had previous sexual contact with men.^130^ This is also true for endometrial cancer due to a significantly higher prevalence of nulliparity and a trend toward obesity.^131^

Transgender and gender non-binary patients require a multidisciplinary approach, which is particularly challenging. One study found that transgender men have 10 times higher odds of having an inadequate Pap smear compared with cisgender women due to androgen therapy-induced cervical and vaginal epithelial atrophy and provider/patient discomfort with the exam.^132^ There is also a lack of guidelines regarding Pap testing in transgender women. One study conducted in the Netherlands that tested neovaginal swabs forHuman papillomavirus (HPV) in this population discovered that 20% of sexually active transgender women tested positive for high-risk HPV compared to 0% of sexually inactive transgender women.

In addition, GC patients who self-identified as Black race compared to White race were 3 times more likely to have SD.^133^

### Previvors

Special attention must be given to previvors of potential cancer, because these high-risk women (“previvors”) struggle with the same treatment-related sexual sequelae but often receive even less attention as they do not have a cancer diagnosis.

#### Evidence

Concerns about sexual side effects can be a reason for non-adherence to hormonal cancer treatments, or—for example, for women with hereditary cancer mutation gene with high risk for breast and ovarian cancer—, a reason for delaying or ignoring recommendations for (risk-reducing) surgery. Studies investigating sexual functioning after risk-reducing salpingo-oophorectomy in women with familial cancer syndromes found that participants often experienced discomfort during sexual intercourse, especially when the surgery was performed in the pre-menopausal years.^134^ Another common complaint in this population is low libido, and HRT has been reported to improve but not alleviate sexual symptoms.^135^

## Conclusions

The SD needs of cancer patients, particularly those with GCs, were not acknowledged and appropriately addressed until several decades had passed. A substantial body of knowledge exists concerning these specific needs, the impediments encountered by patients and HCPs, and compelling data warrant our focused consideration. HCPs should address sexual health issues with patients with gynecologic cancers and their partners before treatment and throughout treatment. A multidisciplinary team should offer appropriate sexual interventions that can improve the QoL of patients and their partners.
